# AIRE deficiency, from preclinical models to human APECED disease

**DOI:** 10.1242/dmm.046359

**Published:** 2021-02-05

**Authors:** Marine Besnard, Francine Padonou, Nathan Provin, Matthieu Giraud, Carole Guillonneau

**Affiliations:** Université de Nantes, Inserm, CNRS, Centre de Recherche en Transplantation et Immunologie, UMR 1064, ITUN, F-44000 Nantes, France

**Keywords:** AIRE, mTEC, APECED, APS-1, Organoid, Knockout model

## Abstract

Autoimmune polyendocrinopathy candidiasis ectodermal dystrophy (APECED) is a rare life-threatening autoimmune disease that attacks multiple organs and has its onset in childhood. It is an inherited condition caused by a variety of mutations in the autoimmune regulator (*AIRE)* gene that encodes a protein whose function has been uncovered by the generation and study of *Aire*-KO mice. These provided invaluable insights into the link between *AIRE* expression in medullary thymic epithelial cells (mTECs), and the broad spectrum of self-antigens that these cells express and present to the developing thymocytes. However, these murine models poorly recapitulate all phenotypic aspects of human APECED. Unlike *Aire*-KO mice, the recently generated *Aire*-KO rat model presents visual features, organ lymphocytic infiltrations and production of autoantibodies that resemble those observed in APECED patients, making the rat model a main research asset. In addition, *ex vivo* models of AIRE-dependent self-antigen expression in primary mTECs have been successfully set up. Thymus organoids based on pluripotent stem cell-derived TECs from APECED patients are also emerging, and constitute a promising tool to engineer *AIRE*-corrected mTECs and restore the generation of regulatory T cells. Eventually, these new models will undoubtedly lead to main advances in the identification and assessment of specific and efficient new therapeutic strategies aiming to restore immunological tolerance in APECED patients.

## Abstract (precis)

**Summary:** In this Review, we discuss the *in vivo* and *in vitro* models available to study AIRE deficiency, and how they may contribute to restoring immunological tolerance in APECED patients.

## Introduction

Preclinical research using experimental animal models of diseases is pivotal to advance the understanding of mechanisms involved in these diseases and to successfully translate bench research to the clinic. The selection of the model and its accuracy remain critical since the success rate of drugs reaching clinical development remains low. Worldwide, there are 7000 rare diseases recognized, affecting more than 350 million people, but only <10% of these diseases have an approved drug treatment (Villalón-García et al., 2020). Clinical trials to evaluate therapeutic candidates for rare diseases are challenging as, by definition, only small groups of patient population are affected.

The autoimmune polyendocrinopathy candidiasis ectodermal dystrophy (APECED; also known as autoimmune polyglandular syndrome type I, APS I) is one of these rare human autoimmune diseases (Perheentupa, 1980). APECED is an autosomal-recessive disorder caused by a mutation in the autoimmune regulator (*AIRE*) gene that is expressed in the thymus and whose protein product, AIRE, is essential for central immune tolerance (Villasenor et al., 2005). AIRE is involved in the expression of tissue-restricted antigens (TRAs), i.e. tissue constituents that are not ubiquitously expressed. These antigens are essential for negative selection as they contribute to the projection of the complete self repertoire at the local site of negative selection and, thus, enable the elimination of all autoreactive T cells. The APECED disease, thus, involves autoreactive T cells that escape deletion, as well as autoantibodies, and leads to premature death in young adults. To date, there is no cure to prevent or treat the APECED syndrome (Kisand and Peterson, 2011).

To better understand the human APECED pathology, AIRE-deficient mouse models have been generated since the identification of causative gene; they have been essential to study and get a better understanding of the APECED disease (Hubert et al., 2009; Mathis and Benoist, 2009). However, flaws remain, since these models only recapitulate limited aspects of human ACEPED pathology and its clinical features and, to the best of our knowledge, these mouse models have not been used to translate therapeutic drug candidates to the clinic.

In this Review, we aim to describe the latest advances in the different APECED models, including a rat model generated by our lab and used to study the disease. Moreover, we discuss new approaches, such as *ex vivo* models and organoids generated from embryonic or induced pluripotent stem cells, to better understand, challenge and assess immunotherapies.

## Human APECED: clinical features and genetic causes

**Fig. 1.**
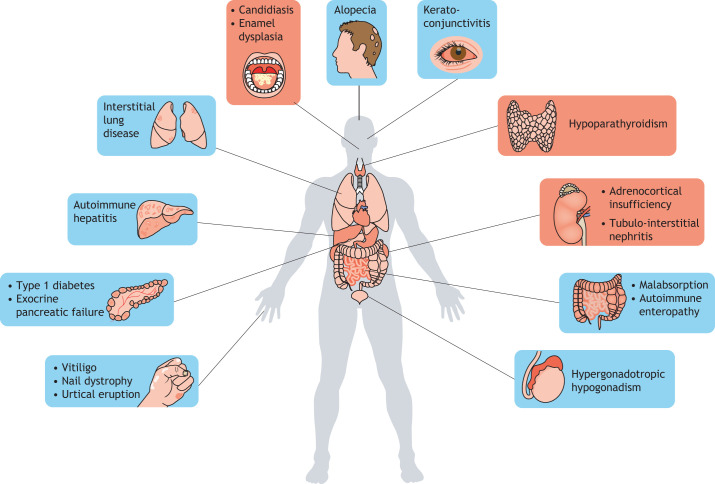
**Common symptoms of APECED.** Representation of different manifestations usually observed in APECED patients, including the historical Whitaker's triad (candidiasis, hypoparathyroidism and adrenocortical insufficiency; red) and the symptoms that have been linked to APECED syndrome only recently (blue).

The first documented case of APECED syndrome was reported in 1929 by Thorpe and Handley, describing of a four-and-a-half-year-old girl suffering from chronic tetany (see Glossary, Box 1), hypoparathyroidism, chronic oral mycelial infection and cornea ulceration (Thorpe, 1929). However, the term APECED is more descriptive of the syndrome and appeared only in 1980 (Perheentupa, 1980). One characteristic of this potentially fatal disease is the incidence of several severe auto-immune lesions within peripheral tissues – not all of which are present in affected individuals – resulting from the central immune tolerance defect. In the past, patients were only diagnosed with APECED if presenting with at least two symptoms of the so-called Whitaker's triad, comprising chronic mucocutaneous candidiasis (CMC), hypoparathyroidism (HP) and adrenal insufficiency (Addison disease, AD) (Box 1) – all three of which are considered to be hallmarks of this disease (Esselborn et al., 1956; Neufeld et al., 1980). More recently, the spectrum of APECED symptoms has been expanded to include ≤30 related manifestations. Amongst others, they include type 1 diabetes (T1D), hypergonadotropic hypogonadism, ovarian failure, hepatitis, keratoconjunctivis, pernicious anemia, malabsorption, alopecia, vitiligo, urtical eruption and enamel hypoplasia (Fig. 1; Box 1) (Ferre et al., 2016; Orlova et al., 2017). APECED patients usually harbor between five and 20 symptoms that, preferentially, appear during childhood; however, some develop with age and without any predictability of severity or diversity (Ahonen et al., 1990; Constantine and Lionakis, 2019; Ferre et al., 2016; Perheentupa, 2006). In many cases, the development of symptoms is preceded by production of specific autoantibodies – another characteristic of the APECED syndrome (Ekwall et al., 1998). Indeed, patients produce a wide array of autoantibodies, some of which correlate with the presence of organ-specific autoimmune manifestations. For example, antibodies directed against cobalamin binding intrinsic factor (CBLIF, also known as GIF), glutamate decarboxylase (GAD; Box 1) and GA-binding protein transcription factor subunit beta 2 (GABPB2) are associated with the development of pernicious anemia, vitiligo and autoimmune hepatitis, respectively (Fishman et al., 2017). However, in contrast to the progressive development of symptoms with age, the antigen repertoire targeted by autoantibodies does not expand. This implies that autoantibodies alone cannot fully explain the accumulation of the APECED-associated autoimmune manifestations (Fishman et al., 2017). Autoantibodies that were found in APECED patients to target cytokines, such as interleukin (IL)-17 and IL-22, have been linked to CMC, whereas autoantibodies targeting type I interferon (type I IFNs) negatively correlate with the incidence of T1D; the latter might, therefore, be of therapeutic interest (Meyer et al., 2016). Anti-IFNω antibodies are highly specific of this pathology, as they are only found in patients diagnosed with a thymoma or with APECED (Burbelo et al., 2010). Moreover, they usually appear before the onset of other clinical manifestations and can be found in all APECED patients. For these reasons, anti-IFNω antibodies are now used as a diagnostic tool for APECED syndrome (Kisand et al., 2008). This discovery fundamentally improved the diagnosis of APECED as, until then, only two diagnostic criteria had been available – the presence of the classic association of symptoms, i.e. those from Whitaker's triad or an *AIRE* mutation. Indeed, the clinical picture has evolved with the discovery of new manifestations and the documentation of prevalent symptoms from Whitaker's triad has declined (Perheentupa, 2002). In addition, since APECED is mostly inherited recessively, scrutiny of the *AIRE* gene by sequence analysis is only done when relatives of the patient are affected or when specific symptoms have developed. However, recent studies have shown that APECED can also be inherited in a dominant manner through mono-allelic missense mutations in the first plant homeodomain (PHD1) zinc finger of AIRE, which then suppress wild-type AIRE in a dominant-negative manner (Cetani et al., 2001; Oftedal et al., 2015). Prevalence of APECED remains relatively low, with an average incidence of 1:90,000–1:200,000 in most European countries (Ferre et al., 2016). However, this strongly increases within an isolated population, such as the Finnish (1:25,000) and Sardinian (1:14,000) or within that of Iranian Jews (1:9000), probably due to a historic founder-mutation effect, specifically the Arg257X, Arg139X and Tyr85Cys mutations (with X representing any amino acid), respectively (Ahonen et al., 1990; Rosatelli et al., 1998; Zlotogora and Shapiro, 1992).

**Box 1. Glossary**

• **Addison disease (AD), also known as primary adrenal insufficiency and hypocortisolism:** an endocrine pathology affecting adrenal glands, resulting in a deficit in steroid hormone synthesis. Symptoms include weight loss, abdominal pain and weakness.

• **Alopecia:** complete or partial loss of hair that can happen on the scalp, i.e. in patches (alopecia areata) or the entire head (alopecia totalis), or over the whole body (alopecia universalis).

• **Aromatic L-amino acid decarboxylase (AADC, officially known as DCC):** an enzyme that catalyzes several different decarboxylation reactions in the biosynthesis of various neurotransmitters and neuromodulators.

• **Chronic mucocutaneous candidiasis (CMC):** chronic *Candida* spp. infection of the mucosa, nails and skin that persists, owing to an immune disorder linked to a T cell defect.

• **Chronic tetany:** a condition characterized by spasms, cramps and overactive neurological reflexes as a result of low calcium blood levels that are often the consequence of hypoparathyroidism (see below).

• **Enamel hypoplasia:** a developmental defect that weakens the surface of teeth, due to defective formation of the hard protective layer covering the outside of the tooth.

• **Epithelial cell adherence molecule (EpCAM):** a common surface marker protein of epithelial cells.

• **Glutamate decarboxylase (GAD):** an enzyme that catalyzes the decarboxylation of glutamate to GABA, the main inhibitory neurotransmitter.

• **Gonadic failure:** a disorder in which testes or ovaries fail to produce either sex hormones or gametes, resulting in fertility issues.

• **Hypergonadotropic hypogonadism (HH), also known as primary or peripheral gonadal hypogonadism:** the defective response of gonads to hormones, caused by problems with the pituitary gland or hypothalamus. It is the result of decreased testosterone or estradiol production, respectively, in males or females, inducing a delay in sexual development and diminished reproductive functions.

• **Hypoparathyroidism (HP):** failure of the parathyroid gland to efficiently produce the parathyroid hormone, *in fine* leading to low blood calcium levels.

• **Immunological tolerance:** capacity of the immune system to recognize the body's own components and not react to them, which is vital for the protection against autoimmune diseases. The tolerance process takes place in the thymus, whose main function is to control thymocyte development, and to discriminate between self- and non-self antigens.

• **Induced pluripotent stem cells (iPSCs):** somatic cells reprogrammed back to an embryonic-like pluripotent state.

• **Keratoconjunctivis:** simultaneous inflammation of the cornea and conjunctiva.

• **Medullary thymic epithelial cells (mTECs):** a population of TECs located in the medulla of the thymus. mTECs express and present a large number of self-antigens to the developing T cell to ensure their education and prevent autoimmune reactions.

• **Protein-disulfide isomerase pancreas specific (PDIp, officially known as PDIA2):** a member of the protein disulfide isomerase (PDI) family, acting as a molecular chaperone that catalyzes the formation of disulfide bonds in secretory proteins.

• **Pernicious anemia:** decrease of red blood cell due to B12 vitamin malabsorption in the intestines.

• **Regulatory T cells (Tregs):** a subpopulation of T cells that help prevent autoimmune manifestations by regulating the activity of immune cells. They control the immune response to and self- and non-self antigens.

• **T cell receptor (TCR) repertoire:** describes the T cell diversity within the immune system of an individual in a physiopathological context and it represents the repertoire of antigens encountered by the TCR.

• **Tissue-restricted antigens (TRAs):** tissue constituents that are not ubiquitously expressed.

• **Type 1 diabetes (T1D):** an autoimmune disorder that affects pancreatic Langerhans islet cells and results in very little to no insulin production.

• **Vitiligo:** a progressive autoimmune disorder affecting the skin that manifests as patchy loss of pigmentation.

### *AIRE* mutations and APECED

To date, 145 *AIRE* mutations, including numerous mutant alleles, have been associated with APECED, from single-nucleotide mutations to large deletions across the gene's entire coding sequence. Although missense mutations of the gene seem to cluster preferentially in the exons that encode the CARD and PHD1 domains, there seems to be no such pattern for insertions or deletions (indels). Most *AIRE* mutations that are not indels occur in the CARD domain; however, the most prevalent mutation, p.R257*, is located in the SAND domain (Stolarski et al., 2006; Trebušak Podkrajšek et al., 2005). It was initially believed that all *AIRE* mutations lead to the autosomal recessive inheritance of APECED; but, recent studies identified dominant *AIRE* mutations in the SAND and PHD1 domains that induce a non-classic form of APECED. This form features very few, if not unique, milder autoimmune manifestations (Ahonen et al., 1990; Cetani et al., 2001; Oftedal et al., 2015), and its discovery suggests that dominant *AIRE* mutations play a previously unrecognized role in the induction of common organ-specific autoimmune disorders (Oftedal et al., 2015).

### AIRE and thymic tolerance

As discussed in more detail below, mouse APECED models in which the *Aire* gene has been inactivated or knocked out revealed that AIRE is specifically expressed in mature medullary thymic epithelial cells (mTECs, see Boxes 1 and 2) of the thymus (Derbinski et al., 2001; Rosatelli et al., 1998). mTECs are characterized by high expression levels of class II major histocompatibility complex (MHC class II) molecules and of a wide array of self-antigens (Danan-Gotthold et al., 2016), which they present to developing thymocytes. In mTECs, AIRE controls the expression of thousands of tissue-restricted antigens (TRAs) that normally are only expressed in one or a few peripheral tissues (Kyewski and Klein, 2006). Although many functional aspects of AIRE remain unknown, it certainly is involved in release of RNA polymerase II pausing at promoters of AIRE-dependent genes (Giraud et al., 2014; 2012) and in recruitment of chromatin-remodeling factors that facilitate transcriptional elongation (Abramson et al., 2010). Additionally, AIRE-dependent gene expression is regulated through a post-transcriptional mechanism that shortens the 3′ untranslated region of AIRE target transcripts to increase their stability (Guyon et al., 2020).

**Box 2. Specific markers and function of TECs**

The role of the thymus in establishing immunological tolerance is based on the functional selection of T cells, a process that is orchestrated by thymic epithelial cells (TECs) (Takahama, 2006). Whereas cortical thymic epithelial cells (cTECs) are involved in thymocyte lineage commitment and in the positive selection of T cells based on the recognition of peptides-MHC molecules (Klein et al., 2014; Takahama, 2006), medullary epithelial cells (mTECs) mediate the negative selection of autoreactive T cells based on the unique ability of mTECs, to express and present tissue-restricted antigens (TRAs) to developing T cells and to eliminate the autoreactive ones (Anderson et al., 2002; Klein et al., 2014; Stritesky et al., 2012). Early markers of cTEC and mTEC lineages include the cytokeratins KRT8 (K8) and KRT5 (K5), respectively (Sekai et al., 2014). Mature mTECs also highly express CD80, MHC-II molecules and the autoimmune regulator AIRE, and are characterized by expressing a high numbers of AIRE-induced TRAs (Gäbler et al., 2007; Sekai et al., 2014).

Impaired expression of AIRE-dependent TRAs in mouse models of APECED impedes the negative selection of developing self-reactive thymocytes (Liston et al., 2003). This has been demonstrated in transgenic mice that have thymocytes harboring T-cell receptors (TCRs) specific to the self-antigen hen egg lysosome (HEL) under the control of the rat insulin promoter (RIP), a promoter that depends on action of AIRE in mTECs (Liston et al., 2003). In contrast to *Aire*-KO mice that do not express HEL in mTECs, few HEL-specific T cells were retrieved from wild-type (WT) mice, showing that AIRE-dependent HEL expression resulted in depletion of T cells able to recognize HEL peptides (Liston et al., 2004; 2003). However, the role of AIRE in shaping immunological tolerance (Box 1) appears to rely not only on the clonal deletion of autoreactive thymocytes but also on the generation of regulatory T cells (Tregs; see Box 1) (Yang et al., 2015). In addition, AIRE is also involved in the mechanisms that enable Tregs to suppress autoimmune manifestations in the periphery of the immune system (Aricha et al., 2011; Teh et al., 2010). These findings show that AIRE plays a key role in the establishment of immunological tolerance, by promoting the negative selection of developing autoreactive thymocytes and by generating Tregs that efficiently suppress autoreactive responses elicited by autoreactive T cells in their periphery. These findings have implications on how to treat APECED patients.

### Current standard of care for APECED patients

Currently, APECED patients receive a combination of treatments tailored to their individual clinical profile. CMC is the most common clinical feature; it requires daily oral medication and close monitoring to avoid chronic *Candida* spp. infection, as it can lead to the development of oral squamous cell carcinoma (Böckle et al., 2010). Normally, CMC is treated with antifungal drugs such as fluconazole, topical ketonazole or amphotericin B for azole-resistant forms (Constantine and Lionakis, 2019; Humbert et al., 2018). APECED patients might also receive hormone replacement therapies comprising synthetic thyroid hormones, mineralo-corticoids, hydrocortisone or sex steroids (Jankowska, 2017; Napier and Pearce, 2012; Winer et al., 2008; Yeap et al., 2016) to treat hormone deficiencies resulting from HP, AD and/or gonadic failure (Box 1). Symptoms that are linked to an excessive response of autoreactive T cells, like autoimmune hepatitis, tubulo-interstitial nephritis or autoimmune enteropathy, are treated with immunosuppressants, such as azathioprine, mycophenolate or corticosteroids (Gentile et al., 2012; Manns et al., 2010; Ulinski et al., 2006). However, the long-term use of immunosuppressive drugs causes significant and unavoidable adverse reactions that can have life-long deleterious effects. For example, corticosteroid therapy in young APECED patients slows their growth and delays puberty (De Leonibus et al., 2016; Polito and Di Toro, 1992). In addition, immune inhibition caused by corticosteroid therapy in children and adults increases their susceptibility to infections – a significant issue in patients already prone to CMC. Hence, there is a profound need to develop more-targeted therapeutics to treat this disease. Recently, rituximab immunotherapy has been used with relative success to treat pneumonitis in the context of APECED; it leads to a clinical improvement without affecting the production of autoantibodies against potassium channel regulatory protein (KCNRG) (Ferré et al., 2019). Nevertheless, as APECED patients are still at risk of premature death (Borchers et al., 2020), their management is very complex and requires the collaboration of numerous specialists, such as dentists, dermatologists, endocrinologists and pediatricians.

The first significant step to improve our knowledge on the APECED syndrome and to evaluate the efficacy of new therapies was achieved only 20 years ago, when scientists generated the first rodent model of AIRE deficiency. We describe its details below.

## Preclinical rodent models of APECED

To study the role of AIRE in the establishment and/or maintenance of immunological tolerance, several rodent models of APECED disease were generated by inactivating *Aire* in mice and rats. Here, we summarize all existing preclinical models of APECED, highlighting their strengths and limitations in relation to human APECED pathology. These models can be used in parrallel to study the heterogeneity and mechanisms underlying the APECED syndrome caused by different known human mutations.

### Mouse APECED models: their strengths and limitations

**Fig. 2.**
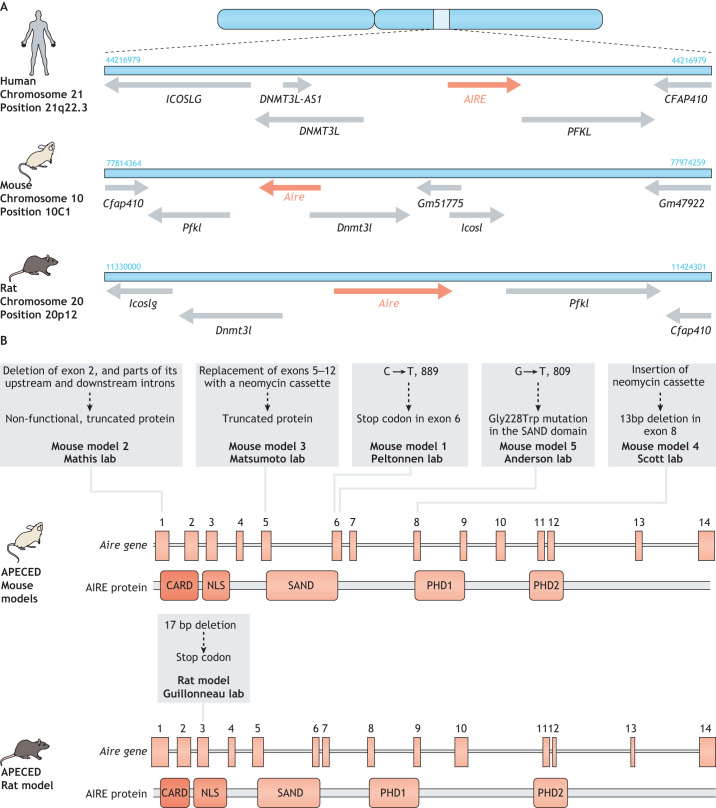
**Comparison of the *AIRE/Aire* locus in human, mouse and rat, and strategies to generate APECED rodent models.** (A) Schematic representation of *AIRE*/*Aire* locus organization in human, mouse and rat, showing the different genetic contexts. (B) Summary of the strategies used to develop APECED mouse and rat models, providing the location of the genetic editing and its consequence for the Aire protein.

The first available mouse models of APECED syndrome were generated from various mouse strains and genetic backgrounds (summarized in Table 1 and Fig. 2) using two main approaches: 1) engineering genetic mutations found in human APECED patients into the murine *Aire* locus and, 2) using exon targeting to delete exons that encode functional domains of Aire (Fig. 2B). The first APECED mouse, which we call model 1, was generated in the Peltonnen lab based on a mutation commonly found in Finnish APECED patients (Ramsey et al., 2002). This mutation corresponds to a cytosine→thymine nucleotide transition at position 889 (C→T, 889) that causes a premature stop codon, thereby truncating exon 6 of the human *AIRE* (Björses et al., 1998). To mimic this mutation, Peltonnen and colleagues designed a construct that targeted exon 6 through homologous recombination, leading to the insertion of a neomycin cassette at the beginning of exon 6 (Ramsey et al., 2002). The APECED mouse model 2, generated by the Mathis lab, uses a shorter *Aire* transcript in which the premature truncation of exon 1 caused the deletion of exon 2 and of some of the upstream and downstream introns, leading to a non-functional AIRE protein (Anderson et al., 2002). The APECED mouse model 3, generated in the Matsumoto lab, was also designed independently of any known human mutation. In this model, a neomycin cassette replaced exon 5 to exon 12 of the *Aire* locus, thus yielding a truncated AIRE protein that lacks a large segment of its functional domain (Kuroda et al., 2005). However, APECED mouse model 4 – containing a common human APECED-associated mutation found in the Anglo-American population (Hubert et al., 2009) – was generated in the Scott lab (Heino et al., 2001), comprising a 13 bp deletion in exon 8 (967–979), which disrupts the PHD1 domain of the protein.

**Table 1:**
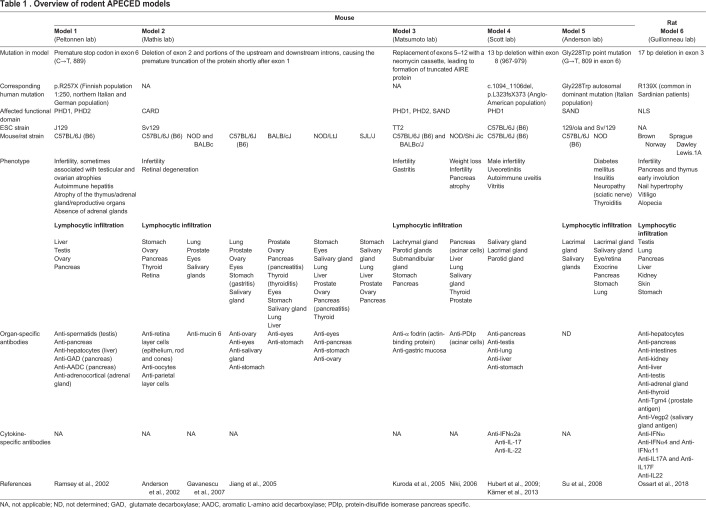
**Overview of rodent APECED models**

The *Aire*-deficient mice described above have a range of infertility problems but present with normal weight and size compared with their littermates, both postnatally and at an age of ∼2–3 months (Anderson et al., 2002; Hubert et al., 2009; Kuroda et al., 2005; Ramsey et al., 2002). Most of their immunological traits, such as T cell proliferation, cytokine production, CD4:CD8 ratio, thymocyte and lymphocyte numbers, expression of differentiation markers, and *in vitro* antigen presentation appear to be normal at birth and up to early adulthood. This is except mouse model 2 (Mathis lab); mice of this model present with increased numbers of activated/memory T cells (CD44^high^CD62L^low^) in peripheral lymphoid organs at the age of ∼2–3 months (Anderson et al., 2002). An increased number of mTECs was also observed in mice of models 1 and 4 at the age of ∼2–3 months (Anderson et al., 2002; Hubert et al., 2009). In models 2, 3 and 4, absence of AIRE is also associated with the loss or significantly reduced expression of several autoantigen genes, thus impairing the negative selection of autoreactive T cells usually mediated by AIRE in the thymus (Anderson et al., 2002; Derbinski et al., 2001; Kuroda et al., 2005; Niki, 2006; Su et al., 2008).

All these mouse APECED models display age-dependent organ lymphocytic infiltration, with variation in the targeted tissues possibly due to environmental factors and genetic backgrounds, as the original APECED mouse models 2 and 3 were back-crossed onto several different genetic backgrounds (Anderson et al., 2002; Gavanescu et al., 2007; Jiang et al., 2005; Kuroda et al., 2005; Niki, 2006). Numerous serum antibodies against different tissues were detected in early adulthood in most *Aire*-deficient mouse models, and the number and frequency of these antibodies progressively increases with age (Anderson et al., 2002; Hubert et al., 2009; Jiang et al., 2005; Kuroda et al., 2005).

Experiments involving thymic chimeras demonstrated that thymocytes derived from *Aire*-deficient mice are autoreactive and can transfer the autoimmune disease when transplanted to immuno-deficient recipients (Anderson et al., 2002; Kuroda et al., 2005). Initially, the number of Tregs (Box 1) and their function seemed to be normal in APECED mouse models 1, 2, 3 and 4, suggesting that only the overproduction of autoreactive T cells induces autoimmune manifestations (Kuroda et al., 2005). However, subsequent studies revealed that *Aire* deficiency also affects the function of Tregs. Indeed, an analysis of the T cell receptor (TCR; Box 1) repertoire of Tregs (CD4^+^Foxp3^+^ and CD8^+^CD28^low^) provided molecular evidence that AIRE is potentially involved in shaping the TCR repertoire of Tregs (Malchow et al., 2013a, 2016; Pomié et al., 2011). Moreover, a comparative analysis of CD8^+^CD28^low^ Tregs from WT and APECED model 4 mice (Hubert et al., 2009) revealed that, despite equal representation and similar immunosuppressive activity, the CD8^+^CD28^low^ Tregs from *Aire*-KO animals fail to control the onset of colitis when using adoptive cell transfer (ACT) in vivo together with colitogenic cells, a phenotypic feature of APECED patients (Pomié et al., 2011) – which is in contrast to CD8^+^CD28^low^ Tregs from WT mice.

Another mouse APECED model, hereafter, referred to as model 5, was developed in the Anderson lab (Su et al., 2008) (Table 1) and is based on an autosomal dominant mutation found in Italian patients, who show a heterozygous base substitution at position 809 of the cDNA sequence (G→T, 809 in exon 6) (Cetani et al., 2001). This nucleotide change leads to replacement of amino acid (aa) glycine with tryptophan at position 228 (Gly228Trp) in the SAND domain of human AIRE. Mice in model 5 present with autosomal dominant autoimmunity and a spectrum of disease manifestations that are different compared to those observed in the other mouse APECED models discussed above. This is because the AIRE protein carrying the Gly228Trp mutation appears to exert a dominant-negative effect that prevents WT AIRE protein to reach active transcription sites in mTECs (Su et al., 2008).

Despite the insights these mouse models provided regarding etiology and pathology of APECED, significant phenotypic and clinical differences exist between *Aire*-deficient mice and human APECED patients. For example, no animal of the APECED mouse models described here displayed the most common, visible autoimmune and ectodermal manifestations of APECED, i.e. CMC, HP and vitiligo – not even those of mouse models bred onto the non-obese diabetic (NOD) genetic background that exhibited a more severe autoimmune phenotype (Gavanescu et al., 2007; Jiang et al., 2005; Niki, 2006; Su et al., 2008). In addition, none of these models had autoantibodies directed against cytokines, such as type I IFNs, IL-22 and IL-17, which are commonly detected in APECED patients. However, a recent study reported that the APECED mouse model 4 (Hubert et al., 2009; Table 1) does have IFNα2a, IL-17 and IL-22-neutralizing autoantibodies (Kärner et al., 2013); and, whereas APECED remains a life-threatening autoimmune disease in humans, *Aire*-deficient mice have a life expectancy that matches that of their WT littermates, despite their organ-specific autoimmunity.

As such, none of these mouse APECED models have been able to recapitulate the severe clinical features seen in APECED patients. They have, nevertheless, provided important insights into the functional relationship between *Aire* and the cellular and molecular pathogenic mechanisms of this disease, enabling the function of AIRE to be investigated in the selection process of T cells and in the establishment of immunological tolerance. All the previously described AIRE-deficient mouse models also played an important role in understanding the role of AIRE in central immune tolerance. However, for proper clinical studies, there is still a need for an animal model that explicitly displays the phenotypical traits of APECED patient. In 2018, a potentially accurate rat model of the disease was designed in the Guillonneau lab (Ossart et al., 2018). The following section presents the strengths and limitations of this APECED rat model.

### The rat model of APECED: strengths and limitations

As Fig. 2A shows, although organization of the *Aire* locus is similar in humans and rats, the murine *Aire* locus overlaps with another gene. As such, disrupting *Aire* in rats might more faithfully recapitulate the clinical features of APECED patients. To the best of our knowledge, only one *Aire*-deficient rat model exists, generated by our own group (Ossart et al., 2018) (Table 1). It was generated by targeting exon 3, which encodes the nuclear localization signal (NLS) sequence of *Aire*, to induce a 17 bp deletion that mimics the human Arg139X mutation commonly found in Sardinian APECED patients (Rosatelli et al., 1998). This mutation leads to an early stop codon, resulting in the premature termination of AIRE translation and reproduces in rats many of the main human characteristics of the APECED syndrome (Ossart et al., 2018). Three lines of these rats were generated by back-crossing the founder *Aire*-deficient Brown Norway rats with WT Sprague-Dawley or Lewis rats for several generations (Table 1). Despite some insignificant differences in terms of symptom severity, the overall phenotype of the rats was similar between each strain (Ossart et al., 2018). This observation supports the hypothesis that the consequences of *Aire* deficiency do not primarily depend on the genetic background but probably more on the layout of the *Aire* locus.

At approximately 6 months of age, animals of all *Aire*-deficient rat strains start to develop skin disorders, including patchy hair loss suggestive of alopecia, depigmentation (vitiligo), and nail overgrowth (nail dystrophy) – symptoms that are frequently seen in APECED patients (Collins et al., 2006). Moreover, several organs, including liver and kidney, show extensive lymphocytic infiltration in all strains of *Aire*-deficient rats, correlating with increased serum levels of alkaline phosphatase and creatinine, respectively (Ferre et al., 2016; Orlova et al., 2017). Both male and female *Aire*-deficient rats show reproductive defects, even when mated with WT animals, and even though testes and ovaries appear to be anatomically normal, thus recapitulating the fertility problems observed in APECED patients (Christin-Maitre et al., 1998; Schaller et al., 2008). In addition, exocrine pancreatic tissue destruction, a major clinical complication in some APECED patients, is a highly prevalent phenotype seen in >90% of *Aire*-deficient rats (Perheentupa, 2006). Overall, histological analyses have revealed that 79% of *Aire*-deficient rats exhibit pancreatic fat accumulation, a decrease in acini, intralobular focal lymphocyte infiltration and hyperplasia of the islets of Langerhans (Ossart et al., 2018). However, glucose blood levels remain normal and they do not develop diabetes, in contrast to APECED patients (Paquette et al., 2010).

Thymopoiesis does occur in *Aire*-deficient rats, with the number and proportion of immune cells being similar to those in WT animals; an exception being decreased numbers of plasmacytoid dendritic cells and natural killer T cells, and increased numbers of effector T cells (Ossart et al., 2018). Transcriptomic comparisons of the thymus between *Aire*-deficient and WT rats and mice demonstrated that, in rats, AIRE does not regulate the expression of the same set of self-antigen genes, possibly explaining the difference in auto-reactivity observed between rodents (Ossart et al., 2018). Additionally, Fezf2 – another key factor involved in inducing the expression of TRAs in mTECs and potentially involved in the establishment of negative selection – is downregulated in the thymus of *Aire*-deficient rats but not in that of *Aire*-deficient mice (Takaba et al., 2015). This suggests that *Aire* deficiency decreases the complexity of the self-antigen repertoire presented in the rat thymus, resulting in the increased escape of autoreactive T cells and a larger array of autoimmune manifestations.

*Aire*-deficient rats also produce a large panel of autoantibodies against several antigens, including those found in the kidney, liver, testis, intestines, adrenal gland and pancreas (Ossart et al., 2018). As in humans, we found no correlation between the titers of these autoantibodies and the severity of the associated symptoms; as such, their importance in the etiology and pathology of APECED remains to be clearly established. Studies in *Aire*-deficient mice have reported the opposite result (DeVoss et al., 2008; Gavanescu et al., 2008), possibly due to the fact that the autoantibody repertoire in mice strongly differs from that of humans suffering from APECED (Pöntynen et al., 2006). In particular, APECED-specific autoantibodies, such as anti-IFNω, anti-IL-17 and anti-IL-22, are not found in most of *Aire*-deficient mouse models, except in model 4 (Kärner et al., 2013), but their levels in *Aire*-deficient rats are comparable to those in APECED patients (Ossart et al., 2018). This absence of spontaneous specific autoantibody production in mouse models of *Aire* deficiency further suggests that the immunopathological mechanisms that occur in these mouse models differ from those of the rat model and of APECED patients. Thus, the *Aire*-deficient rat seems to be an appropriate animal model in which to study autoantibodies in the context of APECED.

Although the rat model of *Aire* deficiency recapitulates many features of the APECED syndrome, the Whitaker's triad of symptoms remains to be observed in these animals. Whether those disparities are linked to *Aire* itself is still unknown. One hypothesis explaining the phenotypic differences between *Aire-*deficient mice and rats, and humans suffering from APECED states that each species has its own specificity in terms of immune system components, such as cytokines, complement system, B cell- and T cell-signaling pathways, γδ T cells, Th1/Th2 differentiation, etc (Mestas and Hughes, 2004). One particular example is that humans produce four subclasses of immunoglobulin G (IgG), i.e. IgG1, IgG2, IgG3 and IgG4, which have no direct homologues in mice and rats. Mice also lack expression of some Fc receptors (FcRs), such as FcαRI, FcγRIIA and FcrγRIIC, all of which play a crucial role in the immune response as they establish a link between adaptive immune cells that produce Igs and innate cells that express FcRs (Bruhns, 2012). Altogether, small divergences might be compounded by the central immune defect due to AIRE deficiency and result, *in fine*, in different phenotypes.

Mice have been extensively investigated for immunological research during the last decades, while the use of rat models for immunology-related investigation is more recent and, still, less frequent. As a consequence, most available techniques and tools are not tailored to rats. Despite this, rat models appear to be very useful as they better represent a number of human diseases, such as Duchenne muscular dystrophy (Ouisse et al., 2019) and, currently, the rat model is the most appropriate for preclinical studies of APECED. We foresee that *Aire*-deficient rat models will also benefit fundamental immunology studies regarding mechanisms of action of AIRE; particularly, because higher numbers of primary mTECs can be obtained from *Aire*-deficient rats as compared with *Aire*-deficient mice, as the availability of these cells is a limiting factor in *ex vivo* experiments. In combination with animal models, these *ex vivo* experiments – which are discussed in more detail below – are a great asset to study thymic mechanisms under pathological conditions.

## *Ex vivo* models to assess mTEC function

Although animal models of APECED are invaluable to understand the events that link AIRE to the negative selection of autoreactive thymocytes and the selection of Tregs, we also need new models to gain further insights into the molecular mechanisms that underlie the mode of action of AIRE. As a result, *ex vivo* models, showing AIRE-mediated induction of TRAs and the impact AIRE has on T cell development, have been generated to investigate such mechanisms. Since primary TECs (see Box 2, Specific markers and function of TECs) die rapidly in standard culture systems *ex vivo*, TEC lines were initially used as *in vitro* systems in which to study induction of gene expression through AIRE. Although these TEC lines provided key findings (Abramson et al., 2010; Giraud et al., 2014), they also have several major limitations, including loss of *AIRE* expression, which has to be restored by transfecting these cells with an *AIRE* expression vector. To model induction of gene expression through AIRE in a more physiologically relevant manner, *ex vivo* models have been set up by using primary TECs in settings that better mimic the complex environment of the thymus, which keeps TECs alive and functional.

To date, only a few *ex vivo* culture systems of primary TECs in a 3D network have been established (Pinto et al., 2013; Villegas et al., 2018). In contrast to previously described two-dimensional (2D) models of TEC cultures (Bonfanti et al., 2010; Kont et al., 2008; Mohtashami and Zúñiga-Pflücker, 2006; Palumbo et al., 2006), 3D culture models preserve mTEC lineage functions, and the cells express TRAs under the control of AIRE and other transcription factors.

### 3D organotypic co-culture

**Fig. 3.**
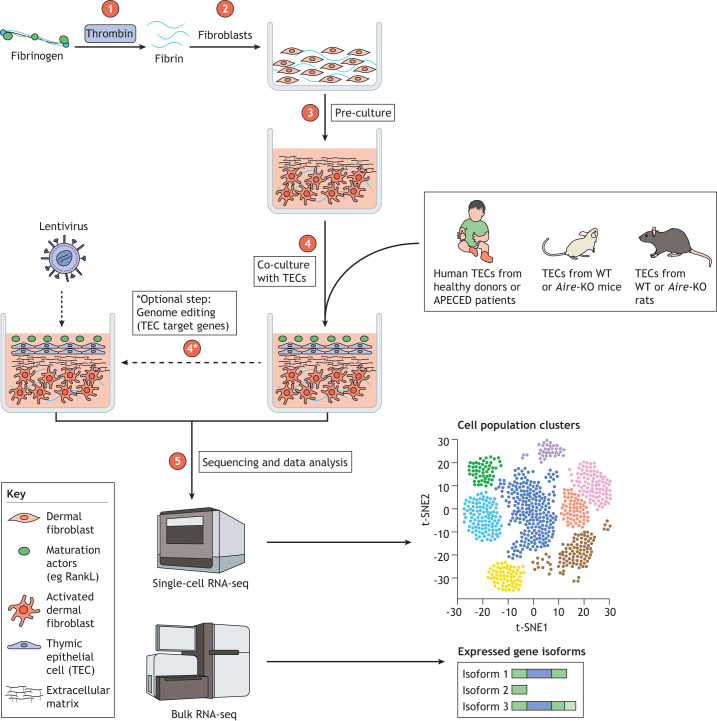
**TEC 3D organotypic co-culture model.** (1) The scaffold of this tissue culture setup is the association of soluble fibrinogen polymerizing into insoluble strands of fibrin through the action of thrombin. (2) Dermal fibroblasts are added to fibrin strands. (3) A few days of pre-culture are required to activate the fibroblasts and to allow them to produce a unique extracellular matrix that is suitable for epithelial cell culture. (4) Freshly extracted thymic epithelial cells (TECs) from different species can be added to the culture together with certain maturation factors, such as the Rank ligand (RankL), which is key for the maintenance of mature and functional TECs. (4*) An optional, additional next step is lentiviral-based gene therapy or gene editing to correct *AIRE* deficiency or to knock down the expression of specific genes relevant to the particular APECED research question. (5) This model can also be used to perform sequencing experiments (bulk or single-cell RNA-seq) to analyze *AIRE*-dependent gene expression or to characterize TEC heterogeneity.

Primary TECs show various biological similarities to keratinocytes in the skin (see Box 3, TECs and keratinocytes). As such, an *ex vivo* 3D organotypic co-culture (OTC) system that supports TEC survival and expansion has been developed, which draws on an *in vitro* model of skin development (Boehnke et al., 2007; Stark et al., 2006). The maturation process of both TECs and keratinocytes depends on their close interaction with stromal cells, such as fibroblasts, and on a 3D structural network of extracellular molecules – the extracellular matrix (ECM) (Depreter et al., 2008; He et al., 2002; Hunziker et al., 2011; Jenkinson et al., 2003; Ulyanchenko et al., 2016). The 3D OTC model mimics dermal tissue by using dermal fibroblasts that are embedded in an inert, semi-solid matrix of insoluble fibrin strands to mimic the ECM (Fig. 3). The addition of TGF-β to this model induces the activation and proliferation of the dermal fibroblasts. Purified mature AIRE-positive mTECs extracted from young (4–6 weeks-old) mice are then seeded onto this matrix within a specific medium that contains the RANK ligand (RankL) (Fig. 3) – reportedly an essential factor for the terminal differentiation of AIRE-positive mTECs (Akiyama et al., 2008; Hikosaka et al., 2008; Rossi et al., 2007). The 3D matrix enables activated fibroblasts to secrete a number of key factors that establish a complex ECM (Fig. 3), which is key for mTEC integrity in this culture model (Boehnke et al., 2007; Stark et al., 2004). In this way, the OTC model preserves key features of mTEC function, such as expression of AIRE and its dependent TRAs, and has, therefore, been instrumental in the identification of the molecular mechanisms that underlie mTEC developmental features, such as the key differences between immature and mature mTECs, as reported for the intact thymus (Pinto et al., 2013). This model could also be used to identify the precise molecular mechanisms that underlie AIRE-dependent expression of TRAs.

**Box 3. TECs and keratinocytes**

Keratinocytes (skin cells) and TECs share many biological similarities (Petrie and Zúñiga-Pflücker, 2007). Indeed, TECs are organized into a 3D network that is crucial for the homeostatic maintenance of the thymic microenvironment and provide an excellent support for the education and maturation of functional thymocytes (Gordon and Manley, 2011). Keratinocytes form a multi-layer, tightly connected sheet that forms the outermost protective layer of the skin (Simpson et al., 2011). Both keratinocytes and TECs express the transcription factor FOXN1, which is necessary for their development and functional integrity (Baxter and Brissette, 2002; Bleul et al., 2006; Gordon and Manley, 2011; Nehls et al., 1996). In the thymus, FOXN1 is required to induce differentiation of both cTECs and mTECs (Gordon and Manley, 2011). In the epidermis, FOXN1 plays an important regulatory role in the development and homeostasis of keratinocytes, and their function in wound healing (Bukowska et al., 2018). Keratinocytes and TECs also express a set of similar cytokeratins (Bonfanti et al., 2010; Cabral et al., 2001; Langbein et al., 2003; Sekai et al., 2014) and differentiation factors, and their progenitors share similar markers, such as PLET-1, RAC1 and SMAD7, which play very important roles in the differentiation of these cells into specific subsets (Depreter et al., 2008; He et al., 2002; Hunziker et al., 2011).

However, although the OTC model provides an optimal environment for *ex vivo* TEC culture, its 3D organization might not be a perfect match for the thymus – it is still a model that sustains viable TECs for ∼1 week.

### Human thymus-derived cell culture

A culture model was designed to allow the expansion of functional TECs from human thymic explants and to address a key problem of earlier versions of this type of model. Here, successive rounds of enzymatic digestion to isolate TECs from other types of thymic cell population (Fernández et al., 1994; Patel et al., 1995; Röpke, 1997; Skogberg et al., 2015) affected the expression of TEC surface molecules and impaired the viability of the cells (Autengruber et al., 2012; Shichkin et al., 2017). In contrast to these earlier approaches, the model reported by Villegas et al. involves an efficient, enzyme-free procedure that enables human primary mTECs to be extracted and, subsequently, expanded from fresh thymic explants (Villegas et al., 2018). Fresh thymic fragments were obtained from immunologically normal human babies (aged 2 days to 1 year) undergoing corrective cardiovascular surgery. Those thymic fragments were cultivated in a medium that supports the migration of various types of thymic cell population (Nancy and Berrih-Aknin, 2005; Nazzal et al., 2014; Wakkach et al., 1996), and the eventual migration and expansion of TECs around the explant. This expansion model was maintained for a few days and the functional properties of mTECs were assessed through expression of AIRE and of AIRE-dependent TRAs. The results of these studies show that the human-derived mTECs of this model retain their ability to secrete important signaling molecules, such as cytokines, chemokines and growth factors, that are essential for the differentiation and maturation of T cell subsets (Cowan et al., 2016; Hauri-Hohl et al., 2014; Kimura and Kishimoto, 2010; Kondo et al., 2019; Lkhagvasuren et al., 2013).

The human thymus-derived culture system is a short-term model that cannot be expanded beyond 7–8 days, which limits the types of study that can be performed (Villegas et al., 2018). Although *ex vivo* models of mTEC culture are a great way to assess mTEC function, they are also dependent on the availability of primary human thymic tissues. Organoid and stem cell-derived models might, therefore, be better suited to expand mTEC differentiation and functional TEC studies.

## Organoids and stem cell-derived models

Thymic organoids are the next step towards a more realistic thymic model that would enable us to study the signals that trigger mTEC differentiation into their mature AIRE-positive state and to carry out T cell differentiation *ex vivo*. A 3D thymic organoid model would need to mimic the thymic microenvironment and have different types of cell population interacting within the ECM that, as discussed above, plays a key role in the survival and development of a thymic cell population. Significant progress in ECM modelling has recently been made using artificial ECMs (Seet et al., 2017) and decellularized tissues (Fan et al., 2015; Hun et al., 2017), which can support the generation of functional T cells *ex vivo* and are expected to greatly benefit research on stem cell-derived thymic models (Seet et al., 2017). Since induced pluripotent stem cells (iPSCs; Box 1) were first developed (Takahashi and Yamanaka, 2006), there has been growing interest in differentiating these cells into functional thymic tissue. Indeed, iPSCs are crucial to develop models with which to study the ontogeny and function of rare types of cell population, like mTECs, which are difficult to isolate and expand *ex vivo*. iPSC-derived cells also have the inherent capacity to harbor genetic diversity, an essential capacity for research in immunology. A key goal of APECED research is to derive iPSCs from the somatic cells of patients and then use gene editing to correct their endogenous *AIRE* gene mutations. The gene-edited iPSCs could then be differentiated into functional mTECs that express the restored AIRE protein and all the AIRE-dependent and -independent TRAs. This approach could result in promising clinical applications, notably cell therapies, where corrected syngeneic mTECs are transplanted to restore the functionality of thymic tissue.

A first step toward this goal has been achieved with the differentiation of mouse embryonic stem cells (ESCs) into EpCAM^+^K5^+^K8^+^ TEC-like cells (Lai and Jin, 2009; Parent et al., 2013; Su et al., 2015), using a 14-day differentiation strategy (see Box 4). Here, two key markers of thymic lineage commitment, *FOXN1* and *HOXA3*, were expressed at similar levels in the resulting cells. After their transplantation into nude recipient mice, these ESC-derived TEC-like cells restored proper thymic organization, as evidenced by the formation of typical medullary and cortical structures. An increase in functional peripheral T cells was also observed, indicative of the transplanted differentiated cells showing normal thymic activity. TECs have also been differentiated from human iPSCs, with comparable results (Chhatta et al., 2019; Inami et al., 2011; Sun et al., 2013). More recently, the transplantation of reaggregated differentiated mouse iPSCs into nude recipient mice was shown to promote the tolerance of skin grafts and the generation of functional T cells (Otsuka et al., 2020). However, several challenges remain that hinder further refinements to this approach. First, the differentiation efficiency achieved by these culturing protocols remains low, with ∼10% of cells expressing the TEC marker epithelial cell adhesion molecule (EpCAM) (Box 1) (Otsuka et al., 2020; Soh et al., 2014), and so further studies are needed to optimize these protocols. Another priority is to develop robust protocols that can be adapted to different iPSC lines, as reproducibility remains a main issue. The difficulty of maintaining TECs in culture also jeopardizes the final stages of iPSC-derived TEC differentiation. Hopefully, recent advances of TEC conservation in culture will make the co-culturing of TECs with T cells possible, in order to support crosstalk between these two cell types and to enable the induction of the cellular programs that lead to their respective maturation. In addition, recent findings have revealed a substantial and unrecognized degree of TEC heterogeneity (Bornstein et al., 2018; Dhalla et al., 2019 preprint). In the past, a relatively small marker set was used to define differentiated TECs, to distinguish few distinct types of TEC population. However, the recent application of single-cell transcriptomics revealed a substantial degree of TEC heterogeneity (Bornstein et al., 2018; Dhalla et al., 2019 preprint), providing us with a more precise way to identify a particular population of iPSC-derived TECs and its signaling pathways. Thus, thymic models based on iPSCs will benefit from new insights provided by single-cell transcriptomics and are expected to closely mimic the biological mechanisms that occur *in vivo.* In addition, over time, the differentiation of APECED patient-derived iPSCs into functional TECs is expected to lead to efficient cell therapies, e.g. transplantable TECs or Tregs obtained from an *ex vivo* T cell development system in which T cell precursors interact with iPSC-generated TECs.

**Box 4. iPSCs – thymic differentiation strategies**

To differentiate iPSC lines derived from somatic cells such as fibroblasts or B cells (Otsuka et al., 2020; Su et al., 2015) into a functional thymic epithelium, cells must replicate the steps of thymic embryonic development, i.e. they must differentiate from definitive endoderm (DE) into anterior foregut endoderm and then into third pouch pharyngeal endoderm (Parent et al., 2013). Several protocols have been established and optimized to generate individual iPSC lines. Generally, DE is induced by culturing iPSCs for 5 days with activin A (INHBA) and, in some cases, with WNT3A and the GSK3 inhibitor CHIR99021 (Otsuka et al., 2020; Parent et al., 2013; Soh et al., 2014; Sun et al., 2013). The anteriorization stage relies on the effect of retinoic acid (RA) combined with that of BMP- and WNT-signal inhibitors, LDN193189 and IWR1, respectively (Inami et al., 2011; Otsuka et al., 2020; Parent et al., 2013; Soh et al., 2014). The TGF-β inhibitors SB431542 or LY364947 are also crucial at this stage. In the final steps of differentiation, cells are usually exposed to BMP4, WNT3A, RA, and FGF signals, such as FGF7, FGF8 and FGF10 (Otsuka et al., 2020; Parent et al., 2013). The sonic hedgehog inhibitor cyclopamine has also been shown to improve thymic differentiation. Since these differentiation protocols are highly susceptible to variability, they still need to be fine-tuned to achieve successful differentiation of the thymic epithelium and should be adapted for each individual iPSC line.

### From experiments to human trials

Gene and cell therapy to correct a mutant *AIRE* gene or to correct AIRE function represent a promising approach to cure APECED. In support of this, similar approaches have been employed to treat other rare diseases, by using CRISPR-Cas9- or adeno-associated virus-based gene therapy to restore the correct expression of mutated genes (see also Gene therapy: The ultimate cure for hereditary disease therapy, 2019). However, in the case of APECED, there are some pitfalls to restoring AIRE expression on a tissue-wide scale, since normal expression of AIRE is restricted to the mTEC lineage. Indeed, it has been shown that some tumor-associated antigens are AIRE-dependent and that immune responses to tumors are stronger in *Aire*-deficient mice (Bakhru et al., 2017; Malchow et al., 2013a,b; Träger et al., 2012), indicating that the widespread expression of a corrected *AIRE* gene in patients could increase the risk of an APECED patient to develop cancer. One study, employing a more targeted approach, indicates how this problem might be addressed (Ko et al., 2010) by using Aire-deficient mice, which only express reduced levels of TRA and, therefore, are more susceptible to TRA-induced experimental autoimmune encephalomyelitis (EAE). Retroviral transduction was then applied to overexpress AIRE *in vitro* in cell lines of thymic medullary or dendritic cell origin, as well as in bone marrow cells. In the cell lines, this approach showed reduced expression of TRAs. However, in bone marrow chimeras that had been generated using the transduced bone marrow cells, elevated expression of TRAs resulted in a delay of the symptomatic onset of EAE (Ko et al., 2010). Transplantation of the thymus from allogeneic sources remains under investigation, but has been successfully performed in pediatric patients with a severe primary immunodeficiency called DiGeorge syndrome, which is characterized by thymic hypoplasia or aplasia (Markert et al., 2010). In this study, sixty patients were transplanted with postnatal allogeneic cultured thymus tissues, resulting in >70% survival, and the successful reconstitution of recipient T cells and T cell function. However, the allogeneic origin of the transplanted thymic tissue might limit its long-term function due to anti-donor immune responses. In addition, only tissue from donors who were less than one year old was used to limit the risk of viral exposure to these immunodeficient patients (Markert et al., 2010). To the best of our knowledge, this approach has not been used to treat APECED patients.

Thanks to developments in tissue-engineering techniques, bioengineered artificial thymus organoids are also being developed with the aim of rejuvenating thymus function. Such organoids have been shown to successfully attract lymphocyte progenitors in nude mice, supporting the generation of a complex T cell repertoire and the induction of donor-specific tolerance (Fan et al., 2015; Tajima et al., 2016). However, thymic organoids will need to also mimic the complexity of a real thymus, which – despite recent advances (Fan et al., 2015) – is yet to be achieved. In addition, concomitant cytokine (such as IL-7 or IL-22) and growth factor (FGF7) therapies might be needed to maintain and promote the proliferation of thymic structures (Berent-Maoz et al., 2012; Dudakov et al., 2012).

Given the broad spectrum of symptoms in APECED, translating findings derived from animal models and from *ex vivo* and *in vitro* experiments to the clinic is a real challenge. To diagnose patients at an early stage is of key importance as it allows therapies to take place before irreversible organ lesions have occurred. In addition, monoclonal antibodies represent a tremendously powerful tool that could be used to target specific effector T cells while preserving Tregs. Indeed, they might represent the next-generation therapies for APECED and would also help to avoid the deleterious, long-term side effects of the immunosuppressive drugs currently used to manage APECED patients (Constantine and Lionakis, 2019). Indeed, we have shown that – for complications after transplantation (i.e. solid-organ rejection and graft-versus-host disease) and in patients with Duchene muscular dystrophy – treatment with anti-CD45RC mAb can restore the balance of Teff-to-Treg cells, inhibit transplant rejection and induce tolerance and, thus, protect against muscle loss in Duchene dystrophy (Boucault et al., 2020; Ouisse et al., 2019; Picarda et al., 2017). Treg cell therapy – either from allogeneic sources or genetically modified to restore their function – also represents a potential treatment (Bezie et al., 2019; Flippe et al., 2019). Moreover, the rat model of APECED could be used to develop such immunotherapies because it allows the visual assessment of disease-associate phenotypes, such as alopecia, weight loss and vitiligo.

Finally, although the absence of AIRE is a feature of APECED disease, significantly decreased levels of AIRE have also been observed in patients suffering from Omenn syndrome or Down syndrome, two disorders characterized by severe immunodeficiency and T-cell-mediated autoimmunity (Cavadini et al., 2005; Giménez-Barcons et al., 2014). This strong correlation between thymic AIRE expression and the susceptibility to a wide range of autoimmune manifestations suggest a ‘dose-effect’ of AIRE that may also provide clues for targeted therapeutics.

## Conclusions

**Fig. 4.**
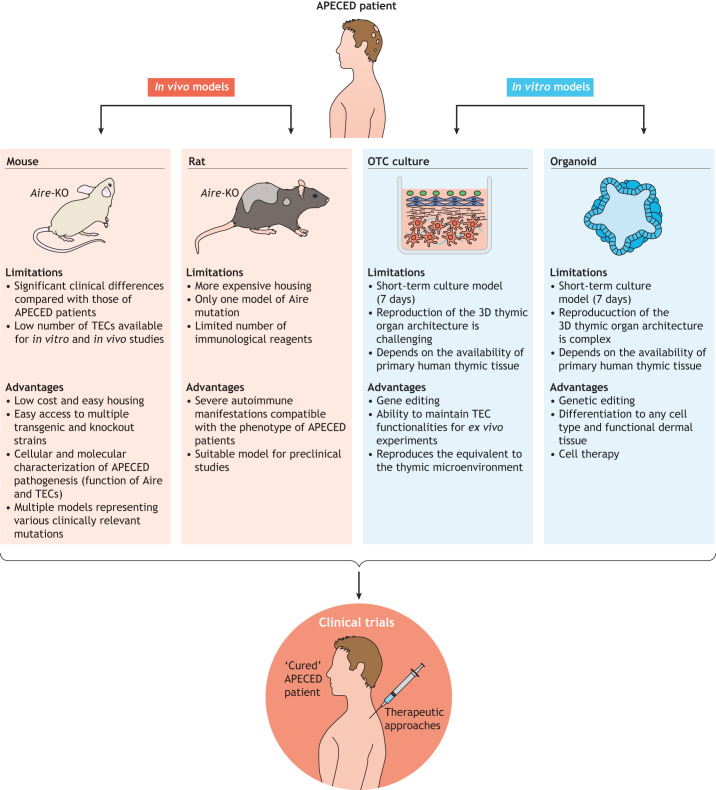
**Research strategies that employ *in vivo* and *in vitro* models of APECED to develop new therapies.** This schematic highlights the advantages and limitations of each model.

The different models generated to study APECED and the development of systems for culturing primary TECs have considerably improved our understanding of the mechanisms that underlie immunological tolerance in the thymus. In addition, they have enabled the development of several pre-clinical therapeutic approaches for controlling autoimmunity in APECED (Fig. 4). However, the existing animal models of APECED do not recapitulate all of the specific aspects of the human disease in humans. Indeed, many mouse models recapitulate only a few aspects of human ACEPED pathology and its clinical features. Moreover, although the rat APECED model can recapitulate several pathological hallmarks of the human disease and will help the translation of drugs to the clinic, additional models are needed, including those of other known *Aire* point mutations that might be associated with specific disease phenotypes. The *ex vivo* 3D OTC and thymus-derived culture models also need to be adapted to the newly discovered broad spectrum of TEC sub-population (Kadouri et al., 2019) the specificities of species origin, i.e. mouse, rat and human. Further studies of these models will undoubtedly offer new insights into function thymic epithelium – notably, with respect to the effect AIRE has across human and murine samples – and will explain the phenotypical differences between APECED in mice, rats and humans. Differentiation of iPSCs into functional thymic tissue will enable functional T cells differentiation *ex vivo*, thereby providing a unique opportunity to restore a dysfunctional immune system through personalized cell therapy treatments. The comprehensive characterization of the complex molecular mechanisms that underlie the effect of AIRE on induction of TRAs, as well as the identification of additional molecular factors involved in the induction of central immune tolerance, will certainly be revealed by using newly developed single-cell transcriptomic and epigenetics approaches. Combined analyses of such new and existing data on TEC biology in human and rodent samples (Bornstein et al., 2018; Kernfeld et al., 2018; Miragaia et al., 2018; Park et al., 2020) are key to tackle TEC heterogeneity and function and, especially, the repertoire of Aire-dependent and independent TRAs. These approaches of deciphering the molecular mechanisms that underlie APECED by using different models and culture system are essential to ensure appropriate and efficient therapeutic measures.
